# Transovarial Transmission of *Babesia ovis* by *Rhipicephalus sanguineus* and *Hyalomma marginatum*

**Published:** 2010-09

**Authors:** GhR Razmi, E Nouroozi

**Affiliations:** Department of Pathobiology, Faculty of Veterinary Medicine, Ferdowsi University, P.O.Box:91775–1793, Mashhad, Iran

**Keywords:** Experimental transmission, *Babesia ovis*, *Rhipicephalus sanguineus*, *Hyalomma. marginatum*, Sheep

## Abstract

**Background:**

*Rhipicephalus sanguineus* and *Hyalomma marginatum* are the most common species in sheep herds in Northeast of Iran. There is preliminary evidence that these species may be the vectors of *Babesia ovis* in Iran. We carried out two experiments in Mashhad area, Khorasan Razavi Province to determine whether *B. ovis* could be transovarially transmitted by *R. sanguineus* and *H. marginatum*.

**Methods:**

In experiment 1, adults of laboratory reared *H. marginatum* and *R.sanguineus* were infected with *B. ovis* isolated from naturally infected sheep in Mashhad area by feeding the ticks on the sheep inoculated intravenously by infected blood samples. The inoculated sheep showed clinical signs with parasitaemia while the adult ticks were engorging on them. The engorged females were collected and kept at 28°C and 85% relative humidity in incubator. Then, larval, nymphal and adult stages derived from engorged females were used to infest the clean sheep. In experiment 2, two splenectomized sheep were infested only with the same adult ticks of two species.

**Results:**

Examination of smears and PCR of blood samples to detect of *B. ovis* in infested sheep in two experiments were negative.

**Conclusion:**

It seems that *R. sanguineus* and *H. marginatum* can not transovarially transmit *B. ovis* in sheep.

## Introduction

*Babesia ovis* is considered highly pathogenic especially in sheep and causes severe infections, which are characterized, by fever, anemia, icterus and hemoglobinuria. Mortality rates in susceptible hosts range from 30% to 50% after field infections or after experimental infection (1). (4). *B. ovis* was reported from Southern Europe, former Soviet States, Northern Africa, Middle East and Asia (1, 2). Among Ixodid tick, *R. bursa* is only known vector of *B. ovis* (1–3). In addition, it seems that other tick species such as, *R. turanicus, R. evertsi. evertsi, H. a.anatolicum,* and *H. a.excavatum* could be vector of *B. ovis* (1).

*B. ovis* occurs in almost all parts of Iran (4, 5) and is highly pathogenic. It causes anemia, icterus and hemoglobinuria in sheep. Mortality rate can reach 25%–30% in sick animals, which do not receive an effective treatment (6). Although, the role of *R. bursa* in transmission of ovine babesiosis has not been examined in Iran, but it has been considered the main vector for both *B. ovis* and *B. motasi* (5, 7). This species can be found only in central and mountainous areas of Iran but not in South and Southeast (8, 9). It seems that other ticks may be important as vector especially outside the distribution area of *R. bursa*. The prevalence of *B. ovis* infection in Northeast of Iran is high and *R. sanguineus* and *H. marginatum* are dominant ticks among sheep flocks (10, 11).

There is some evidence that *R. sanguineus* and *H. marginatum* are potential vector of *B. ovis* in Mashhad area (10). The aim of the present study was to demonstrate whether *R. sanguineus* and *H. marginatum* could transmit *B. ovis* to sheep*.*

## Material and Methods

### Ticks

The *R. sanguineus* and *H. marginatum* ticks used in this study were originally collected from the field in Mashhad area and were reared in the laboratory condition in the School of Veterinary Medicine, Mashhad, 2007. The batches of each species were fed for several generations on rabbits and sheep without any transmission of *Babesia* spp. to sheep.

### Sheep

The sheep of a local breed with 1 to 2 year of age were purchased from a center of sheep culture without history of babesiosis. The peripheral blood was examined on successive days to ensure that all sheep were free from blood parasite prior to feeding the infected ticks on them.

### Parasite

From April to September 2006, first, blood smears were prepared from suspected sheep to babesiosis and stained by Geimsa method. When the *B.ovis* was microscopically detected in the blood smears, blood samples were collected into EDTA and heparin -coated tubes from infected sheep for PCR and inoculation to sheep.

### PCR and semi nested PCR

For reconfirmation of diagnosis, the blood samples of infected sheep were tested by PCR technique. Total DNA was extracted from EDTA blood samples using a DNA isolation kit (CinnaGene Inc, Iran) according to manufacturer's instructions. Then, the PCR and semi nested PCR was performed according to the method of Shayan et al. (12) for reconfirmation of diagnosis. Briefly, total DNA was extracted from EDTA blood samples using a DNA isolation kit (CinnaGene Inc. Iran) according to manufacturer's instructions. Then, the PCR and semi nested PCR was performed as stated earlier (12).

Briefly**,** four oligonucleotide PCR primers were used to detect *B. ovis* for the PCR and nested PCR. Forward primer (P1): 5′-cacagggaggtagtgacaag-3′, and the reverse (p2): 5′-aagaatttcacctatgacag-3′were used in the first amplification reaction. Internal primers used in a nested PCR were forward primer (P2) 5′-aagaatttcacctatgacag-3′ and reverse primer (P3) 5′-gtctgcgcgcggcctttgcg 3′. The 25 ml reaction mixture contained: 2 µl of template DNA, 2.5 µl of 10× PCR buffer (CinnaGene Inc, Iran), 1 µl MgCl2, 0.5 µl of 200 mM of each dNTP mix (CinnaGene Inc,Iran), 0.2 µl (1unit) of Tag DNA polymerase (CinnaGene Inc,Iran) and 1.25 µl (10 pmol) of each P1 and P2 primers (CinnaGene Inc,Iran) for PCR and dd H2O (16.3µl) were added up to 25 µl. The amplification conditions for *B. ovis* included with an initial enzyme activation at denaturation at 95°C for 5 min, 36 cycles with denaturing at 94°C for 45 s, primer annealing at 54-58°C for 45 s and extension step at 72°C for 45s, followed by final extension at 72°C for 10 min. PCR Products were then chilled at 4°C. The final PCR products were subjected to electrophoresis in a 1.5% agarose gel with TBE buffer. Seminested PCR was performed with the PCR product isolated from the agarose gel using a DNA isolation kit (CinnaGene Inc, Iran). Briefly, the DNA bands were cut from the gel under UV light and dissolved according to the manufacturer's instructions in the binding buffer at 60°C. One to five µl of the eluted DNA was amplified with the primers P2 and P3 using the mentioned method. Samples positive for *B. ovis* produced visible bands at 389–402 bp in the first PCR and 186 bp in the second PCR.

### Experimental transmissions

Three sheep were inoculated intravenously with heparinized blood that infected with *B. ovis*. Simultaneously, 50 pairs of adult *R. sanguineus* and *H. marginatum* ticks put into cotton bags on ears of sheep. After one week, engorged ticks were collected and kept 28°C and 85% relative humidity in incubator. All stages of next generations of the female ticks were used for transmission of *B. ovis*. In the first experiment, 1000 larvae, 500 nymphs and 100 adults were placed on individual sheep. The larval and nymphal of *R. sanguineus* and *H. marginatum* did not complete their development on sheep. In Second experiment, only 100 of adult *R. sanguineus* and *H. marginatum* were placed on the splenectomized sheep. The clinical signs of every sheep have been daily checked for two months. Simultaneously, EDTA blood samples and thin blood smears were collected for PCR (12) and microscopic examination. When the results of two methods for detection of *B. ovis* were negative, the sheep were considered as non-infected animal.

## Results

The isolate of *B. ovis* showed to be highly virulent in all inoculated sheep. They developed typical babesiosis with parasitaemia 2% to 4%. After a prepatent period ranging from 3 to 4 days. The presence of the *B. ovis* was confirmed in blood smears and by using PCR.

All infected sheep died after 7–8 days of apparent clinical signs. The batches of adult of *R. sanguineus and H. marginatum* were engorged during feeding on ear of sheep. In the first experiment, only adult stages of *R. sanguineus and H.marginatum* were attached and were engorged on the sheep.

Clinical sign and parasitaemia were not observed two months after infestation. Thus, the results of PCR on blood samples of infested sheep were not negative at the end of the study. In the second experiment, not clinical symptoms, nor febrile response were observed in splenectomized sheep. Microscopical examinations of blood smears and PCR were also negative after two months post-infestation.

## Discussion

*R. sanguineus and H. marginatum* are two ticks, dominants in sheep flocks in Northeast, Iran (10, 11). This is the first study to evaluate the transmittability of *B. ovis* by *R. sanguineus and H. marginatum.*

The results of this study indicate that any stage of these species cannot transovarially transmit the *B. ovis*. So far, two reports have been published about potential vector of *R. sanguineus and H. marginatum* for *B. ovis*. In our recent study (10) the kinetes of *Babesia* spp. were observed in haemolymph and egg smears of *R. sanguineus* and *H. marginatum* collected from infected sheep with *B. ovis*. Shayan et al. (12) identified *B. ovis* in salivary glands of *R. bursa, R. turanicus and R. sanguineus* by using PCR and semi-nested PCR. The results of first experiment also showed that only adult stages of *R. sanguineus and H. marginatum* could be engorged on the sheep. It seems that the sheep is not a suitable host for feeding of immature stages of *R. sanguineus* and *H. marginatum*. The biology of *R. sanguineus* and *H. marginatum* indicated that immature stages infest birds, rodents and other small mammals, but adults usually parasitize larger animals (13–15).

In conclusion, *R. sanguineus and H. marginatum* cannot transovarially transmit *B. ovis* to sheep and other species such as *R. bursa and R. turanicus* may play a role in transmission of *B. ovis* in sheep in Mashhad area.
